# Hemodynamic Response to Massive Bleeding in a Patient with Congenital Insensitivity to Pain with Anhidrosis

**DOI:** 10.1155/2018/9593458

**Published:** 2018-06-03

**Authors:** Yuki Sugiyama, Sayako Gotoh, Masatoshi Urasawa, Mikito Kawamata, Koichi Nakajima

**Affiliations:** ^1^Department of Anesthesiology and Resuscitology, Shinshu University School of Medicine, Japan; ^2^Division of Anesthesia, Shinonoi General Hospital, Japan

## Abstract

A patient with congenital insensitivity to pain with anhidrosis (CIPA) underwent revision of total hip arthroplasty under general anesthesia with only propofol. During surgery, neither elevation of stress hormones nor hemodynamic changes associated with pain occurred; however, when blood was rapidly lost, compensatory tachycardia was observed. Although patients with CIPA are complicated with autonomic disturbance due to dysfunction of postganglionic sympathetic fibers, this compensatory response indicated that the adrenal glands in patients with CIPA secrete catecholamine as part of a compensatory response during bleeding under general anesthesia.

## 1. Introduction

Congenital insensitivity to pain with anhidrosis (CIPA) is a rare autosomal recessive disease that is characterized by unexplained fever, anhidrosis, and loss of pain sensation [1, 2]. Patients with CIPA frequently suffer recurrent episodes of wounds and bone fractures, which need surgical treatment. For the anesthetic management of patients with CIPA, the control of body temperature, analgesia, and treatment of dysfunction of the postganglionic sympathetic fibers are important. Massive bleeding occurred in a patient with CIPA who underwent revision of total hip arthroplasty under general anesthesia. Here, we discuss the compensatory hemodynamic change during massive bleeding and secretion of stress hormones associated with pain in the patient with CIPA.

## 2. Case Presentation

A 36-year-old woman (height, 147 cm; weight, 50 kg) with CIPA was scheduled for revision of left total hip arthroplasty. She was diagnosed as having CIPA because of recurrent episodes of unexplained fever, anhidrosis, burns, and bone fractures after birth. She had previously undergone 7 operations for spinal deformity and 1 operation of total hip arthroplasty in both the left and right sides. Although lack of general diaphoresis and thermal nociception were observed, the patient performed body surface cooling at her own discretion when she felt she was at a risk of hyperthermia, and her body temperature was kept approximately 36°C. No signs of mental retardation or orthostatic hypotension were observed. No abnormality was detected on chest radiographs and electrocardiograms. Blood biochemistry revealed no abnormality except mild anemia indicated by a hemoglobin level of 10.6 g/dl.

**Figure 1:**
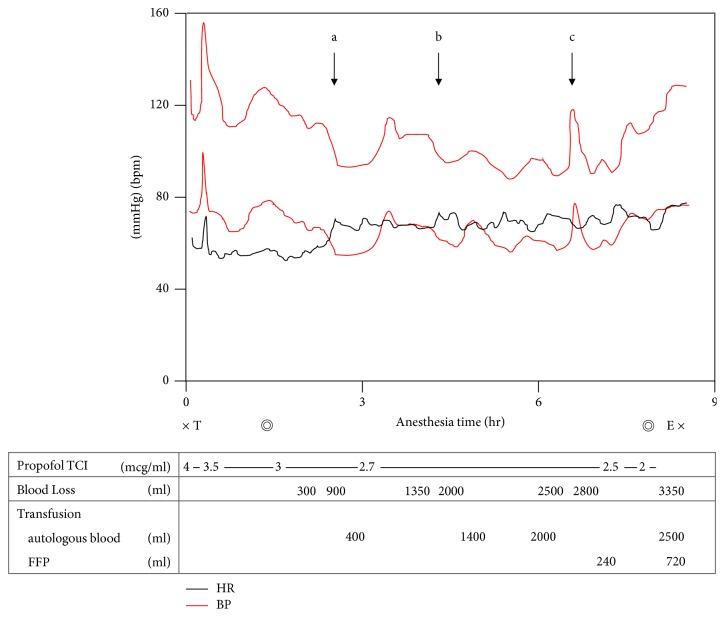
Anesthetic record. The black line indicates heart rate (HR) and the red lines indicate blood pressure (BP). a: six hundred mL of blood loss within 20 minutes, b: six hundred and fifty mL of blood loss within 30 minutes, c: administration of 0.1 mg phenylephrine, ×: start or end of anesthesia, *◎*: start or end of the surgery, T: tracheal intubation, E: extubation, TCI: target-controlled infusion, and FFP: fresh frozen plasma.

No premedication was administered. After the patient was brought into the operating room, routine monitoring and measurement of the bispectral index (BIS) were started. Body temperature was measured at 3 different sites (urinary bladder, esophagus, and precordial skin) and controlled by a hot-air-type heater. Propofol was administered at an effect-site concentration of 4 *μ*g/ml by target-controlled infusion. After muscle relaxation had been achieved by administration of 50 mg of rocuronium, the trachea was intubated. Immediately after endotracheal intubation, systolic blood pressure increased from 130 to 145 mmHg, and heart rate increased from 60 to 95 beats per minute (bpm). Two minutes later, systolic blood pressure had decreased to 125 mmHg. Propofol was continuously infused intravenously at a target concentration of 2 to 4 *μ*g/ml (Figure 1) and BIS levels were maintained between 40 and 60. After an arterial catheter had been placed, her position was changed from the supine to right lateral position. Surgery was then started.

Since no circulatory change associated with pain occurred during surgery, opioids were not administered. Regarding hemodynamics, when 600 ml of blood was rapidly lost within 20 minutes, blood pressure decreased from 113/66 to 93/55 mmHg and heart rate increased from 55 to 70 bpm (Figure 1 a). Similarly, when 850 ml of blood was lost within 30 minutes, systolic blood pressure decreased from 108/65 to 95/60 mmHg and heart rate increased from 66 to 74 bpm (Figure 1 b). Administration of 0.1 mg of phenylephrine increased blood pressure from 87/55 to 117/76 mmHg and decreased heart rate from 70 to 65 bpm (Figure 1 c).

The operative time was 6 hours and 49 minutes, and the duration of anesthesia was 8 hours and 41 minutes. The volume of blood loss was 3350 ml. Blood transfusion was performed with 1600 ml of preoperatively donated autologous blood, 900 ml of salvaged blood, and 720 ml of fresh frozen plasma. Intraoperative body temperature was controlled and kept between 36.0°C and 36.9°C at all 3 measurement points. After surgery had been completed, the patient was returned to the supine position and she was extubated. Since she did not complain of any pain after the surgery, no analgesic was administered. She was discharged at 6 weeks after the operation.

Blood samples were collected 3 times: before anesthesia induction, after the start of surgery, and at the end of surgery. The levels of catecholamine fractions and cortisol were measured. Norepinephrine levels were below the normal range at all time points, and the levels of epinephrine and cortisol were within the normal ranges at all time points (Table 1).

## 3. Discussion

CIPA is caused by a loss-of-function mutation of the TRKA gene, which encodes the receptor of the nerve growth factor (NGF) tyrosine kinase. Without this receptor, primary afferent neurons and postganglionic sympathetic fibers, which are dependent on NGF for growth and survival, are lost, and their loss induces both autonomic disturbance and loss of pain sensation [1, 2]. In anesthetic management of patients with CIPA, attention should be paid to the use for analgesics, abnormal body temperatures due to abnormal sympathetic nervous activity, and their hemodynamics.

There have been some case reports on patients with CIPA who received general anesthesia without any analgesics [3–5] and in whom intraoperative levels of catecholamine and cortisol were measured [6]. In that previous study, levels of catecholamine and cortisol were not elevated by pain stimuli during surgery under general anesthesia. In our case, in which anesthesia was managed only with propofol, the levels of catecholamine and cortisol were also not elevated by pain stimuli (Table 1); however, it has been pointed out that catecholamine levels might not be elevated by stress induction because of the loss of postganglionic sympathetic fibers in patients with CIPA [7].

Recently, the ability of catecholamine secretion in patients with CIPA has been examined using a standing test [8]. In patients with CIPA, blood norepinephrine concentrations have been reported to be below the normal range [7, 8]. Normally, norepinephrine is mainly secreted by sympathetic nerve terminals and the brain, and epinephrine is mainly secreted by the adrenal medulla. When healthy volunteers were upright, normal blood pressure was maintained by norepinephrine. In contrast, when patients with CIPA were upright, blood pressure was maintained by epinephrine, not by norepinephrine [8]; therefore, this response is considered to be a compensatory response by the adrenal medulla in patients with CIPA. As for cortisol, it has been reported that cortisol secretion in patients with CIPA can be increased by stress from limited water intake [7]. The results of those studies suggest that patients with CIPA can secrete epinephrine and cortisol in response to stress.

In our case, a change in hemodynamics was not observed at the start of surgery (Figure 1), and both catecholamine and cortisol levels were low (Table 1), even though general anesthesia was maintained only by propofol. This indicated that the pain stimuli caused by incision did not induce catecholamine or cortisol secretion. In contrast, compensatory tachycardia was observed during bleeding (Figure 1a, b). Unfortunately, we could not obtain both a blood sample during massive bleeding and a control blood sample just before the bleeding, which was difficult to predict; however, this response was considered to be induced by epinephrine secreted from the adrenal grand, as mentioned above [8]. From these findings, we considered that our patient had the ability to secrete epinephrine from the adrenal grand under general anesthesia when the patient was exposed to some stress, and the response after the start of surgery indicated that pain stimuli did not induce any stress in our patient.

Cortisol level usually increases continuously during surgery [9] and reaches a peak after extubation [10, 11]. In our case, cortisol level was within the normal range before anesthesia and decreased to less than the normal range after the start of surgery (Table 1). This decrease might have represented circadian rhythm or a decrease of mental stress after induction of general anesthesia. Although the cortisol level was slightly increased at the end of surgery, the level was close to the lower limit of the normal range and we thought that this small increase in cortisol level was not induced by strong stress but might have been due to the decreased administration of propofol at the end of surgery. These changes in hormones and the capability of hormone secretion suggested that pain stimuli during surgery did not induce any stress in our patient.

Hemodynamic changes in our case were observed at intubation and at administration of phenylephrine followed by bradycardia caused by baroreflex. The pharynx and larynx are innervated by glossopharyngeal and vagus nerves, and baroreflex is regulated by the vagus nerve [12]. These physiological responses occur because these cranial nerves are intact in patients with CIPA. A previous study showed that tachycardia within 10 minutes after induction occurred in 31% of CIPA patients and that the patients could respond to airway manipulation [13]. Intraoperative bradycardia was also observed in 2.8% of CIPA cases [13]. In pediatric patients, cardiovascular complications were reported to be common [3] and to sometimes induce hemodynamic change by an unpleasant sensation [3, 14]; therefore, attention should be paid to hemodynamics in patients with CIPA, even if a compensatory response is present.

## Figures and Tables

**Table 1:** Levels of catecholamine fractions and cortisol.

	**Before anesthesia **	**After the start of surgery**	**End of surgery**	**Normal range**
**Epinephrine**	46	33	9	< 100 (pg/mL)
**Norepinephrine**	37	26	5	100 - 450 (pg/mL)
**Cortisol**	19	5.2	8.1	6.2 - 19.4 (mcg/dL)
